# Lacosamide-Responsive Paroxysmal Tonic Spasms Associated With a New Lateral Cerebral Peduncle Lesion in Multiple Sclerosis: A Case Report

**DOI:** 10.7759/cureus.105347

**Published:** 2026-03-16

**Authors:** M'hamed Riad Amanallah, Hamza Benzakour, Yasmina Zakaria, Khaoula Balili, Mohamed Chraa, Nissrine Louhab

**Affiliations:** 1 Neurology, Centre Hospitalier Universitaire (CHU) Mohammed VI, Faculté de Médecine et de Pharmacie de Marrakech, Marrakech, MAR

**Keywords:** case report, cerebral peduncle, lacosamide, multiple sclerosis, paroxysmal tonic spasms

## Abstract

Paroxysmal tonic spasms (PTS) are abrupt, brief, involuntary, and often painful muscle contractions commonly seen in demyelinating disorders such as multiple sclerosis (MS) and neuromyelitis optica spectrum disorder (NMOSD). These movements are frequently associated with new demyelinating lesions in the corticospinal tract. First-line treatment is usually carbamazepine, a sodium channel blocker, but intolerance and side effects may limit its use. We report a case of a 31-year-old woman with clinically stable relapsing-remitting MS who developed sudden, painful spasms of the left upper limb. MRI of the brain showed a new lesion in the lateral part of the right cerebral peduncle, consistent with her symptoms. She was first started on carbamazepine, which was discontinued due to gastrointestinal side effects. Lacosamide, another antiepileptic with a similar mechanism of action, was introduced at a dose of 50 mg twice daily, resulting in rapid and complete resolution of her symptoms. During follow-up, the patient remained symptom-free and clinically stable. This case highlights the occurrence of PTS secondary to a lateral cerebral peduncle lesion. Lacosamide was highly effective in controlling symptoms, suggesting it is a good alternative for patients who cannot tolerate carbamazepine. Awareness of PTS and timely imaging can guide adequate therapy and improve patients' quality of life. Lacosamide may represent an effective and well-tolerated alternative when first-line therapy cannot be used.

## Introduction

Paroxysmal tonic spasms (PTS), also referred to as paroxysmal dystonias, are sudden, involuntary, and brief contractions of limb and/or trunk muscles lasting less than 1 min, frequently associated with pain and abnormal posturing, classically seen in central demyelinating disorders such as multiple sclerosis (MS) and neuromyelitis optica spectrum disorder (NMOSD) [1]. These spasms occur in the clinical course of these conditions and often correlate with new demyelinating lesions on the corticospinal tract [2]. Although a beneficial effect of pulse steroid therapy for a recently active lesion cannot be excluded, management of PTS is mainly symptomatic. Antiepileptic drugs are highly effective, particularly the voltage-gated sodium channel inhibitor carbamazepine, which is generally considered the treatment of choice [3].

We report a case of a patient with MS who developed paroxysmal tonic spasms secondary to a newly identified lesion in the lateral cerebral peduncle, who showed dramatic improvement with lacosamide. This report highlights the clinical presentation, diagnostic workup, and therapeutic approach, with a focus on the effectiveness of the antiepileptic drug lacosamide, which is another voltage-activated sodium channel inhibitor that was used as an alternative to carbamazepine.

## Case presentation

A 31-year-old female with a history of clinically stable relapsing-remitting multiple sclerosis presented with paroxysmal tonic spasms of her left upper limb. She was diagnosed in 2023 and met the 2017 McDonald criteria, with optic neuritis, MRI lesions in the periventricular and subcortical white matter typical of multiple sclerosis, and cerebrospinal fluid showing oligoclonal bands. The patient was receiving disease-modifying therapy with rituximab, administered as periodic infusions; however, she had missed her most recent scheduled dose prior to the onset of symptoms. On examination, her Expanded Disability Status Scale (EDSS) score was 0, with only mild paresthesias, similar to her last evaluation three months earlier.

Four days before being admitted, the patient began experiencing sudden, painful contractions of the left upper limb, associated with abnormal posturing, sometimes so severe that it led to falls. The movements were suggestive of paroxysmal tonic spasms. Each episode lasted approximately 10-20 s, occurred up to 10 times a day, and caused her significant distress.

**Figure 1:**
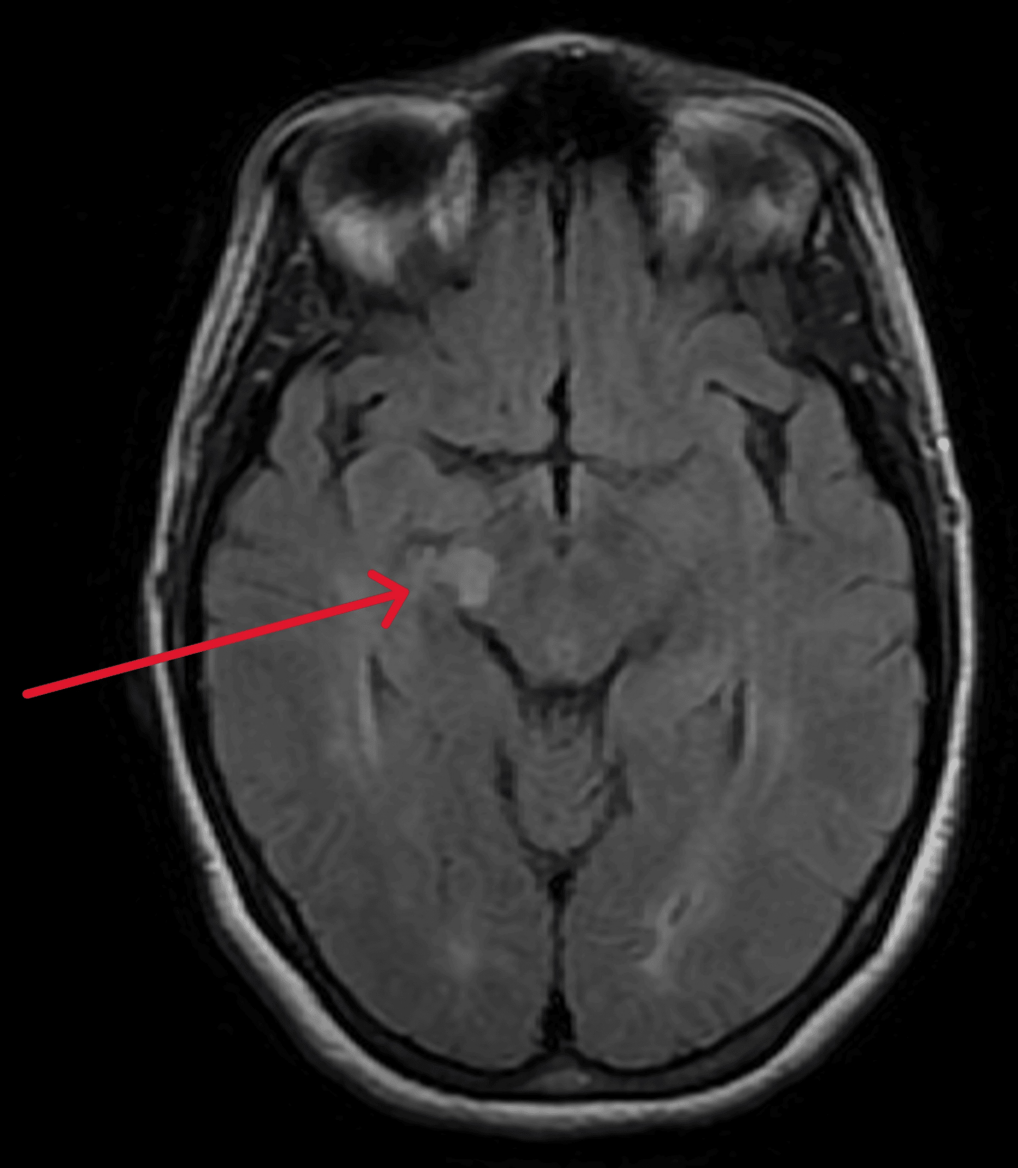
Axial T2-FLAIR MRI demonstrating a new hyperintense lesion in the lateral part of the right cerebral peduncle. The arrow indicates the demyelinating lesion located in the lateral cerebral peduncle on MRI. FLAIR: fluid-attenuated inversion recovery

**Figure 2:**
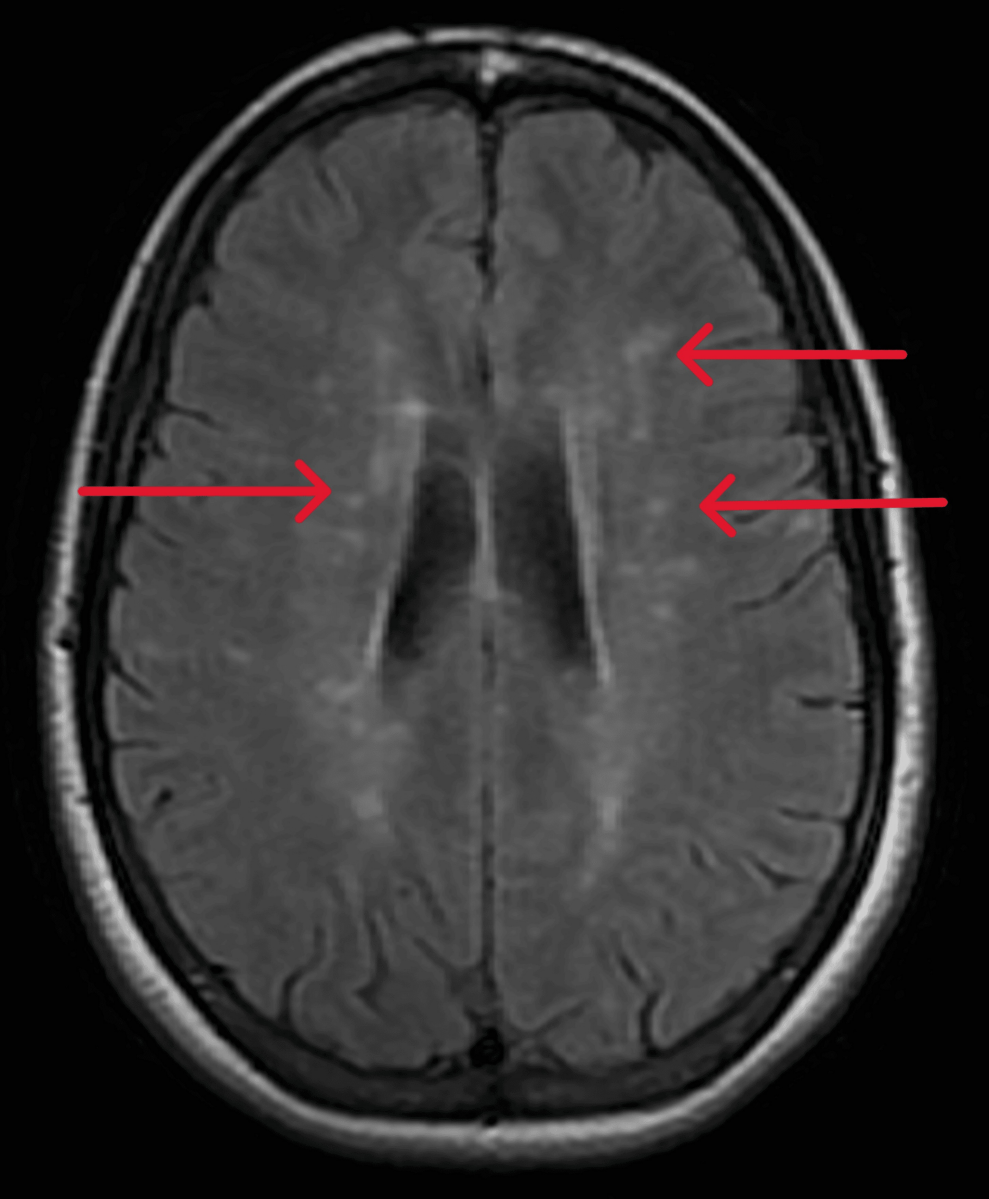
Axial T2-FLAIR MRI demonstrating hyperintense white matter lesions characteristic of multiple sclerosis, stable compared to the previous MRI. The arrows highlight the periventricular lesions of multiple sclerosis (MS). FLAIR: fluid-attenuated inversion recovery

Neurological examination between episodes revealed preserved strength and reflexes, with no new sensory deficits. MRI of the brain revealed a new demyelinating lesion in the right lateral cerebral peduncle (Figure 1), concordant with the distribution of the tonic spasms, along with lesions of the brain white matter that were stable compared to her previous MRI (Figure 2). A spinal MRI was also performed and showed no demyelinating lesions.

An electroencephalogram was normal with no epileptiform discharges. The patient was initially prescribed carbamazepine; however, she developed severe nausea and vomiting and was unable to tolerate the medication. She was subsequently started on lacosamide 50 mg twice daily, which led to dramatic improvement, reducing the spasms from 10 episodes per day to none.

The patient was admitted for further management. She continued lacosamide and received a course of intravenous pulse steroids for the new lesion seen on MRI. During follow-up, the tonic spasms did not recur, and she remained clinically stable on lacosamide therapy.

## Discussion

Paroxysmal tonic spasms (PTS) are well-recognized manifestations of demyelinating disorders, mainly multiple sclerosis (MS) and neuromyelitis optica spectrum disorder [1,2]. These movements are abrupt, stereotyped, brief, and often painful, with dystonic posturing. Reported in 11-15% of patients with MS, PTS represents the most commonly described movement disorder in MS after tremor [4,5]. PTS may occur multiple times per day. Episodes often last less than one minute, and may gradually increase in frequency, are precipitated by movement, and may be preceded by a sensory aura [6]. The pathophysiology is thought to result from ephaptic transmission and hyperexcitability of partially demyelinated axons, leading to abnormal spread of electrical impulses [4].

PTS are classically attributed to lesions affecting the corticospinal tract, although associations with lesions in the thalamus and cerebellum have also been hypothesized. They may therefore potentially be generated at any location along the motor pathway [7,8]. In our patient, MRI revealed a new lesion in the lateral part of the cerebral peduncle, anatomically consistent with the contralateral upper limb involvement. The cerebral peduncle contains descending corticospinal fibers, and focal demyelination at this level likely explains the observed paroxysmal motor manifestations.

The lateral portion of the cerebral peduncle also contains the corticopontine fibers, particularly the temporopontine and occipitopontine projections. Although the clinical presentation and imaging findings strongly suggest that the patient’s PTS resulted from focal hyperexcitability of corticospinal fibers within the lateral cerebral peduncle, it is conceivable that disruption of adjacent corticopontine fibers may have contributed to abnormal motor output. Demyelination or inflammation at this level may reduce inhibitory control from the cerebellum, basal ganglia, or cortical interneurons, thereby allowing abnormal motor bursts to occur. Some authors have suggested that sustained dystonia may be related to dysfunction of the cortico-ponto-cerebello-thalamo-cortical loop [9]. However, the precise role of these fibers in generating paroxysmal movements remains uncertain; further neurophysiological or tractography studies would be required to clarify their contribution to PTS.

The differential diagnosis for paroxysmal tonic spasms includes focal seizures. Focal seizures are usually associated with abnormal epileptiform discharges, particularly during or immediately after an event. The absence of such findings, along with the stereotyped, brief, and painful nature of the spasms, supports the diagnosis of MS-related paroxysmal tonic spasms rather than seizure activity.

Although high-dose corticosteroids are indicated in cases of acute inflammatory activity in MS and may contribute to clinical improvement, symptomatic therapy remains the cornerstone of PTS management. Antiepileptic drugs, particularly carbamazepine, often lead to complete cessation of episodes and are considered first-line treatment [3]. The proposed mechanism involves stabilization of hyperexcitable demyelinated axons and reduction of aberrant neuronal firing. Agents such as gabapentin or oxcarbazepine may also be used, sometimes in combination with baclofen, pregabalin, levetiracetam, or other medications as second-line options [10].

In the present case, carbamazepine was stopped due to significant gastrointestinal side effects. Lacosamide, another voltage-gated sodium channel inhibitor that enhances slow inactivation of sodium channels and stabilizes the resting state of hyperexcitable axons, was introduced as an alternative and resulted in excellent clinical outcomes. The patient experienced complete resolution of spasms shortly after initiation, with sustained benefit during follow-up. The mechanism of action of lacosamide, a slow inactivator of sodium channels, differs slightly from that of carbamazepine, which primarily enhances fast inactivation, and this may explain lacosamide’s efficacy and favorable tolerability in patients who cannot use first-line therapies. This response suggests that lacosamide is a reasonable therapeutic option for patients intolerant to carbamazepine or in whom first-line therapy is contraindicated.

Reports on the use of lacosamide for MS-related paroxysmal tonic spasms remain limited. However, given its mechanism of action and generally favorable tolerability profile, it may be particularly useful in this setting. Baheerathan et al. reported a case of lacosamide-responsive tonic spasms in a 44-year-old woman with neuromyelitis optica spectrum disorder [11]. Paroxysmal movement disorders in demyelinating diseases can present as tonic spasms or kinesigenic dyskinesia-like events. A case of MS-related paroxysmal kinesigenic dyskinesia responsive to lacosamide has also been reported, further supporting the efficacy of lacosamide for paroxysmal motor phenomena in demyelinating disorders when first-line therapy cannot be used [12]. Our observation supports the concept that targeting sodium channel-mediated hyperexcitability is central to symptom control in paroxysmal manifestations of demyelinating disease.

This case highlights two important clinical points. First, the occurrence of PTS in a patient with otherwise stable MS should prompt evaluation for new demyelinating lesions along the corticospinal tract, including brainstem structures. Second, lacosamide may be considered an effective alternative in patients who do not tolerate carbamazepine for symptomatic treatment. Further studies are needed to better define the role of lacosamide in this context. However, this study adds to the evidence supporting its use in paroxysmal symptoms associated with demyelinating disease.

## Conclusions

This case shows that paroxysmal tonic spasms can arise from a new lateral cerebral peduncle lesion in patients with otherwise stable multiple sclerosis. Lacosamide has been highly effective in controlling symptoms in our patient, highlighting its potential as an alternative to carbamazepine in patients who are intolerant or have contraindications. The occurrence of tonic spasms in patients with multiple sclerosis should prompt repeat MRI evaluation to assess for new demyelinating lesions and timely initiation of appropriate therapy.
